# Increased cancer incidence risk in type 2 diabetes mellitus: results from a cohort study in Tyrol/Austria

**DOI:** 10.1186/1471-2458-14-1058

**Published:** 2014-10-10

**Authors:** Willi Oberaigner, Christoph Ebenbichler, Karin Oberaigner, Martin Juchum, Hans Robert Schönherr, Monika Lechleitner

**Affiliations:** Department of Clinical Epidemiology of the Tyrolean State Hospitals Ltd, Cancer Registry of Tyrol, TILAK GmbH, Anichstrasse 35, Innsbruck, Austria; Department of Internal Medicine I, Medical University Innsbruck, Anichstrasse 35, 6020 Innsbruck, Austria; Department of Internal Medicine, University Teaching Hospital Hall, Hall, Austria; Department of Internal Medicine, Saint Vincent Hospital Zams, Zams, Austria; Department of Internal and Geriatric Medicine, Hochzirl Hospital, Zirl, Austria

**Keywords:** Cancer, Type 2 diabetes, Cohort study, Registry

## Abstract

**Background:**

Recent meta-analyses revealed an association between type 2 diabetes mellitus (T2DM) and cancer. The strongest relationship was demonstrated for liver and pancreatic cancer, followed by endometrial cancer. We aimed at assessing the association between T2DM and cancer specifically for Tyrolean patients.

**Methods:**

We investigated cancer incidence in Tyrolean subjects with T2DM by linking the data from the Diabetes and the Cancer Registries. 5709 T2DM patients were included and the sex- and age-adjusted standardized incidence ratio (SIR) was calculated, cancer incidence in the Tyrolean population serving as the standard. Endpoints were the time at which cancer was diagnosed, death or end of the observation period (31 December 2010).

**Results:**

Site-specific analyses revealed statistically significantly elevated SIRs for cancer of the pancreas (1.78, 95% CI 1.02, 2.89) and corpus (1.79, 95% CI 1.15, 2.66) for women, and cancer of the liver (2.71, 95% CI 1.65, 4.18) and pancreas (1.87, 95% 1.11, 2.96) for men. Sub-analyses performed according to the time of diabetes diagnosis revealed that SIR was highest in the first year after diabetes diagnosis, but SIR was demonstrated to be elevated in women for cancer of the liver (SIR 3.37, 95% CI 1.24, 7.34) and corpus (SIR 1.94, 95% CI 1.09, 3.20) and in men for liver (SIR 2.71, 95% CI 1.40, 4.74) in the period more than five years after diabetes diagnosis. In addition, increased risk at borderline statistical significance was observed in females for liver cancer (SIR 2.40, 95% CI 0.96, 4.94) and cervical cancer (SIR 1.81, 95% CI 0.87, 3.32) and in males for kidney cancer (SIR 1.65, 95% CI 0.99, 2.57).

**Conclusion:**

This study revealed a higher risk for cancer at certain sites in Tyrolean patients with T2DM. However, it is important to interpret the results with great caution due to inherent methodological problems. Optimized care programs for patients with T2DM should be integrated into the recommended procedures for cancer screening.

## Background

Diabetes mellitus and cancer are both common health problems and an association between these two conditions was demonstrated by several epidemiological studies. Large meta-analyses have evaluated the increased risk of distinct cancer entities. An about twofold increased risk for patients with type 2 diabetes mellitus (T2DM) was found for liver cancer (2.01, 95% confidence interval (CI) 1.61, 2.51 [1]), pancreatic cancer (1.94, 95% CI 1.66, 2.27 [2, 3]) and endometrial cancer (2.10, 95% CI 1.93, 3.24 [4]), and a less than twofold increased risk for breast cancer (1.20, 95% CI 1.12, 1.28 [5]), colorectal cancer (1.27, 95% CI 1.21, 1.34 [6]), kidney cancer (1.42, 95% CI 1.06, 1.91 [7]), bladder cancer (1.24, 95% CI 1.08, 1.42 [8]) and non-Hodgkin's lymphoma (1.19, 95% CI 1.07, 1.32 [9]). The risk for T2DM patients to develop prostate cancer was demonstrated to be reduced (0.84, 95% CI 0.76, 0.93 [10]), and data about the relation between diabetes mellitus and various other cancer sites like gastric cancer did not provide conclusive results [11–13].

The underlying mechanisms responsible for the increased risk for certain cancer types in T2DM seem to be complex and remain the matter of debate. Growing evidence suggests that obesity, insulin resistance and hyperinsulinaemia are main risk factors for cancer [2, 4, 14]. Hyperinsulinaemia is associated with an increased cancer incidence and progression [15]. The tumor growth-promoting role of hyperglycaemia seems to be dependent on the presence of insulin resistance and hyperinsulinaemia [15, 16]. Finally, certain T2DM diabetes mellitus therapies are discussed with respect to their relationship to cancer risk in T2DM [17].

From the epidemiological point of view, a number of serious methodological problems have been discussed like latency periods, lead time bias, reverse causality, ascertainment bias and competing risks [18]. In consequence, especially results from observational studies have to be interpreted with great caution.

To the best of our knowledge, no epidemiological studies on the relationship between diabetes mellitus and cancer have been conducted in the Central European region. In Tyrol, the Cancer Registry Tyrol (CRT) prospectively collects all incident cancer cases since year of diagnosis 1988 and the Diabetes Registry Tyrol (DRT) registers diabetes mellitus patients attending out-patient departments in Tyrol since 2005. Cancer incidence rates in Tyrol are in the range of rates in Central European countries (with the exception of high prostate cancer rates and high melanoma rates). Completeness of overall incidence and site-specific incidence has been shown to be high (≥ 97%) [19]. The Tyrolean population of about 700 000 is very stable with only minor out-migration, which facilitates cohort studies. As Tyrol is, to the best of our knowledge, the only population in Central Europe covered by a diabetes registry, we felt it would be valuable to investigate the association between diabetes and cancer incidence. Therefore, bearing in mind the great clinical importance of cancer risk for diabetes patients in the interest of optimizing care for diabetic patients and developing standardized control procedures, we aimed to assess the association between T2DM and cancer specifically for Tyrolean patients. Our study findings could serve as a model for the situation in Central Europe and could support cancer screening recommendations for the local T2DM population.

## Methods

The DRT was established in 2005 and registers all patients with type 1 diabetes mellitus, T2DM and gestational diabetes mellitus, who are attending the out-patient departments of hospitals in Tyrol. We collected all newly diagnosed diabetes patients since 2005 as well as all prevalent diabetes patients who attended an out-patient department since 2005 for whatever reason. For the latter group we attempted to retrospectively assess the date of diabetes diagnosis. However, a non-negligible group of patients could not remember their date of diagnosis. Therefore, all patients collected by the DRT to date (N = 7627) can be divided into a group of patients with registered date of diabetes diagnosis (N = 5709, 75% of all patients) and a second group of patients without registered date of diagnosis. Compared to international prevalence estimates, the DRT covers about 15% of all prevalent diabetes patients in Tyrol.

Patient registration is performed using a standardized questionnaire covering basic data on patient characteristics and data on out-patient visits. The questionnaire includes age, sex, body height and body weight, type of diabetes mellitus, diabetes mellitus treatment, HbA_1c_, frequency of severe hypoglycaemia, physical exercise and late complications of diabetes mellitus (nephropathy, retinopathy, neuropathy, foot complications, amputation, myocardial infarction, apoplexy, peripheral arterial insufficiency, coronary artery bypass/PTCA). Patient registration is done on a pseudonymised basis. The pseudonymisation process permits linkage of data for a specific patient registered in different departments and guarantees data confidentiality, because pseudonymisation prevents identification of the patient.

The CRT was established in 1986. Cancer data for the Tyrolean population have been registered on a population basis since 1988. Registration is performed from a standardized questionnaire including sex, age at diagnosis, cancer site and histology, date of diagnosis, stage and basic information on primary cancer treatment. The CRT routinely assesses patient survival status in a passive way. We employ a probabilistic record linkage method to combine incidence data and the official mortality data for Tyrol collected by Statistics Austria. A detailed analysis of completeness and validity of data items has been published [19]; incidence data since year of diagnosis 1988 have been accepted for publication in Cancer Incidence in Five Continents.

The cohort we analyzed was defined as all patients registered by the DRT with defined date of diabetes diagnosis. In order to assess cancer diagnosis for these patients, record linkage between DRT and CRT was performed after pseudonymising CRT data with the same algorithm as applied in the DRT. We linked DRT patients to all invasive cancer sites except non-melanoma skin cancer (NMSC) diagnosed in the time period 1988 to 2010. Like in most cancer registries, collection of NMSC data in Tyrol has not been complete and therefore these data were excluded; death certificate only (DCO) cases were excluded, too. For patients with multiple cancer diagnoses, only the chronologically last cancer diagnosis was included in the analysis in order to weaken reverse causality by study design as far as possible.

We calculated age-standardized incidence ratios treating diabetes mellitus patients as a cohort. Entry date was date of diabetes diagnosis (1st of January of the respective year, because only year of diagnosis is registered). If this date was before 1988, entry was determined to be 1 January 1988 because cancer data were not registered before 1988. Every patient with cancer diagnosis except NMSC was counted as an event, patients who died before cancer diagnosis or who were alive on 31 December 2010 without cancer diagnosis were analyzed as censored. The number of expected cancer cases was computed given the cancer risk in the general population as standard, after adjusting for sex, age in 5-year age groups and period in 5-year period groups. SIR was calculated in the classical way by observed cases divided by expected cases for all cancer sites combined and for specific cancer sites. Confidence intervals were computed by applying an exact method [20].

In order to determine whether a priori fixed design rules influenced our results, we performed a sensitivity analysis by 1) taking the chronologically first cancer diagnosis instead of the last cancer diagnosis and 2) defining a cohort of all patients registered in the DRT (N = 7627), with or without registered date of diabetes diagnosis. The population in Tyrol was 707 485 inhabitants in 2010, of which 51.1% were females. All statistical analysis was conducted using STATA statistical software, version 11 [21].

## Results

We analyzed a cohort of 5709 T2DM patients with well-defined date of diabetes diagnosis, all of whom were treated in out-patient departments of Tyrolean hospitals. Of these patients, 2685 were female (24 145 person years) and 3024 were males (25 697 person years). Mean age at onset was 60 and 55 for females and males, respectively. Mean HbA_1c_ measures were 7.7%, 38.5% of patients showed late complications. Further details on patient characteristics are shown in Table 1.

**Table 1:** **Patient characteristics for T2DM patients**

	Patients with registered date of diabetes diagnosis (N = 5709)	Patients without registered date of diabetes diagnosis (N = 1918)	Full analysis (N = 7627)
Median age at onset (years) and 25 and 75 percentiles	57 48/66	64 55/73	59 50/68
Sex,% females	47.0	42.4	45.9
Distribution by year of diabetes diagnosis (quinquennium)	1988-1990: 10.7%		1988-1990: 8.0%
	1991-1995: 7.3%		1991-1995: 5.5%
	1996-2000: 19.2%		1996-2000: 14.3%
	2001-2005: 25.5%		2001-2005: 19.1%
	2006-2010: 37.3%		2006-2010: 53.1%
HbA_1c_(%) ^1)^	7.7	7.8	7.7
Obesity,% of patients with BMI ≥ 30	41.4	39.1	40.8
Family history,% of patients with diabetes in first-degree relatives	44.8	44.6	44.8
Late complications,% of patients with at least one late complication^2)^	38.5	42.9	38.5

^1)^Data are presented as mean of measures per patient.

^**2)**^ **Late complications:** nephropathy, retinopathy, neuropathy, foot complications, amputation, myocardial infarction, apoplexy, peripheral arterial insufficiency, coronary artery bypass (PTCA).

Age-standardized cancer incidence rates for the most recent period were 259 and 332 for 100 000 females and males, respectively. Median age at diagnosis of cancer patients was 66 years for females and 66 for males, 19%/13% were younger than 50 years and 19%/13% older than 80 years for females and males, respectively. The most common cancer sites among women were breast cancer (28%), colorectal cancer (12%), lung cancer (7%) and melanoma (6%) and among men prostate cancer (28%), lung cancer (14%), colorectal cancer (11%), stomach cancer (5%) and melanoma (5%). More details on cancer incidence, especially age-standardized incidence rates for Tyrol and for many cancer registries worldwide, have been published elsewhere [22, 23].

For female diabetes mellitus patients, 234 cancer diagnoses were assessed. In the site-specific analysis we found a statistically significantly elevated SIR for pancreatic cancer (SIR = 1.78, 95% CI 1.02, 2.89) and corpus cancer (SIR = 1.79, 95% CI 1.15, 2.66). Increased risks, although not statistically significant, were also observed for liver and cervical cancer. Details are shown in Table 2.

**Table 2:** **Cancer risk for T2DM patients by sex and cancer site**

		Females			Males		
Cancer site		Obs	SIR	95% CI	Obs	SIR	95% CI
Head & Neck	[C00-C14; C30-C32]	2	0.40	(0.05, 1.46)	14	0.75	(0.41, 1.26)
Stomach	[C16]	10	1.07	(0.51, 1.97)	8	0.55	(0.24, 1.08)
Colorectum	[C18-C21]	27	0.94	(0.62, 1.36)	44	1.11	(0.81, 1.49)
Liver	[C22]	7	2.40	(0.96, 4.94)	20	2.71	(1.65, 4.18)
Pancreas	[C25]	16	1.78	(1.02, 2.89)	18	1.87	(1.11, 2.96)
Lung	[C33-C34]	15	0.79	(0.44, 1.30)	45	0.92	(0.67, 1.24)
Melanoma	[C43]	9	0.82	(0.37, 1.55)	11	0.70	(0.35, 1.25)
Breast	[C50]	72	1.13	(0.88, 1.42)			
Breast postmenopausal	[C50]	70	1.18	(0.92, 1.49)			
Cervix uteri	[C53]	10	1.81	(0.87, 3.32)			
Corpus uteri	[C54]	24	1.79	(1.15, 2.66)			
Ovary	[C56]	7	0.63	(0.25, 1.29)			
Prostate	[C61]				83	0.85	(0.68, 1.05)
Bladder	[C67]	4	0.89	(0.24, 2.29)	16	1.10	(0.63, 1.79)
Kidney	[C64-C65]	10	1.45	(0.70, 2.67)	19	1.65	(0.99, 2.57)
Brain	[C47; C70-C72]	0	0	(0.00, 1.18)	3	0.73	(0.15, 2.13)
Thyroid	[C73]	8	1.65	(0.71, 3.25)	1	0.30	(0.01, 1.67)
Haem&Lymph^1)^	[C81-C85; C88; C90-C96; D45-D47]	26	1.28	(0.84, 1.87)	21	0.78	(0.48, 1.18)
All cancer sites except NMSC	[C00-C97; D45-D47; exc. C44]	234	1.05	(0.92, 1.20)	291	0.93	(0.82, 1.04)

^1)^Neoplasms in the haematologic/lymphatic system.

For male patients, a total of 291 cancer diagnoses were assessed. The site-specific analysis showed a statistically significantly elevated SIR for liver cancer (SIR = 2.71, 95% CI 1.65, 4.18) and pancreatic cancer (SIR = 1.87, 95% CI 1.11, 2.96) and borderline significance for kidney cancer (SIR = 1.65, 95% CI 0.99, 2.57). Details are shown in Table 2.

Sub-analysis performed according to the time elapsed since diabetes diagnosis revealed that for females (Table 3), in the first year after diabetes diagnosis, SIR was statistically significantly elevated for pancreatic cancer (SIR = 9.51, 95% CI 3.82, 19.59), postmenopausal breast cancer (SIR = 1.99, 95% CI 1.00, 3.57) and for cervical cancer (SIR = 6.22, 95% CI 1.69, 15.92). No statistically significant increased SIR was found during the period two to five years after diabetes diagnosis, while in the period beyond five years after diabetes diagnosis SIR was elevated for liver cancer (SIR = 3.37, 95% CI 1.24, 7.34) and corpus cancer (SIR = 1.94, 95% CI 1.09, 3.20). For male patients (Table 4) we observed statistically significantly elevated SIR for kidney cancer (SIR = 4.64, 95% CI 1.51, 10.83) in the first year after diabetes diagnosis and for liver cancer at 3.02 (95% CI 1.21, 6.21) and 2.71 (95% CI 1.40, 4.74) in the periods two to five years and beyond five years after diabetes diagnosis, respectively.

**Table 3:** **Cancer risk for female T2DM patients by cancer site and follow-up period**

		Follow-up period											
Cancer site		Total			1st year			2 - 5 years			≥5 years		
		Obs	SIR	95% CI	Obs	SIR	95% CI	Obs	SIR	95% CI	Obs	SIR	95% CI
Head & Neck	[C00-C14; C30-C32]	2	0.40	(0.05, 1.46)	0	0.00	(0.00, 8.14)	0	0.00	(0.00, 2.30)	2	0.69	(0.08, 2.51)
Stomach	[C16]	10	1.07	(0.51, 1.97)	1	1.18	(0.03, 6.58)	3	1.01	(0.21, 2.94)	6	1.09	(0.40, 2.36)
Colorectum	[C18-C21]	27	0.94	(0.62, 1.36)	4	1.58	(0.43, 4.05)	7	0.77	(0.31, 1.59)	16	0.93	(0.53, 1.51)
Liver	[C22]	7	2.40	(0.96, 4.94)	1	4.07	(0.10, 22.70)	0	0.00	(0.00, 4.12)	6	3.37	(1.24, 7.34)
Pancreas	[C25]	16	1.78	(1.02, 2.89)	7	9.51	(3.82, 19.59)	4	1.48	(0.40, 3.79)	5	0.90	(0.29, 2.11)
Lung	[C33-C34]	15	0.79	(0.44, 1.30)	2	1.18	(0.14, 4.27)	4	0.66	(0.18, 1.69)	9	0.80	(0.37, 1.52)
Melanoma	[C43]	9	0.82	(0.37, 1.55)	1	0.93	(0.02, 5.19)	3	0.81	(0.17, 2.35)	5	0.81	(0.26, 1.88)
Breast	[C50]	72	1.13	(0.88, 1.42)	12	1.94	(1.00, 3.39)	21	0.98	(0.60, 1.49)	39	1.08	(0.77, 1.48)
Breast postmenopausal	[C50]	70	1.18	(0.92, 1.49)	11	1.99	(1.00, 3.57)	20	1.02	(0.62, 1.58)	39	1.14	(0.81, 1.55)
Cervix uteri	[C53]	10	1.81	(0.87, 3.32)	4	6.22	(1.69, 15.92)	2	0.96	(0.12, 3.47)	4	1.42	(0.39, 3.65)
Corpus uteri	[C54]	24	1.79	(1.15, 2.66)	4	3.14	(0.86, 8.05)	5	1.13	(0.37, 2.64)	15	1.94	(1.09, 3.20)
Ovary	[C56]	7	0.63	(0.25, 1.29)	1	0.95	(0.02, 5.31)	1	0.27	(0.01, 1.51)	5	0.78	(0.25, 1.81)
Bladder	[C67]	4	0.89	(0.24, 2.29)	0	0.00	(0.00, 9.71)	1	0.70	(0.02, 3.92)	3	1.12	(0.23, 3.29)
Kidney	[C64-C65]	10	1.45	(0.70, 2.67)	1	1.57	(0.04, 8.74)	4	1.79	(0.49, 4.59)	5	1.24	(0.40, 2.90)
Thyroid	[C73]	8	1.65	(0.71, 3.25)	2	3.86	(0.47, 13.95)	3	1.69	(0.35, 4.94)	3	1.17	(0.24, 3.42)
Brain	[C47; C70-C72]	0	0.00	(0.00, 1.18)	0	0.00	(0.00, 12.58)	0	0.00	(0.00, 3.55)	0	0.00	(0.00, 2.05)
Haem&Lymph^1)^	[C81-C85; C88; C90-C96; D45-D47]	26	1.28	(0.84, 1.87)	3	1.65	(0.34, 4.83)	5	0.78	(0.25, 1.82)	18	1.49	(0.88, 2.35)
All except NMSC	[C00-C97; D45-D47; exc. C44]	234	1.05	(0.92, 1.20)	42	1.96	(1.42, 2.66)	61	0.82	(0.63, 1.06)	131	1.03	(0.86, 1.23)

^1)^Neoplasms in the haematologic/lymphatic system.

**Table 4:** **Cancer risk for male T2DM patients by cancer site and follow-up period**

		Follow-up period											
Cancer site		Total			1st year			2 - 5 years			≥5 years		
		Obs	SIR	CI	Obs	SIR	CI	Obs	SIR	CI	Obs	SIR	CI
Head & Neck	[C00-C14; C30-C32]	14	0.75	(0.41, 1.26)	1	0.55	(0.01, 3.07)	6	0.94	(0.35, 2.06)	7	0.67	(0.27, 1.37)
Stomach	[C16]	8	0.55	(0.24, 1.08)	1	0.75	(0.02, 4.21)	4	0.85	(0.23, 2.18)	3	0.35	(0.07, 1.02)
Colorectum	[C18-C21]	44	1.11	(0.81, 1.49)	6	1.72	(0.63, 3.74)	14	1.11	(0.60, 1.86)	24	1.03	(0.66, 1.53)
Liver	[C22]	20	2.71	(1.65, 4.18)	1	1.55	(0.04, 8.66)	7	3.02	(1.21, 6.21)	12	2.71	(1.40, 4.74)
Pancreas	[C25]	18	1.87	(1.11, 2.96)	3	3.67	(0.76, 10.73)	7	2.33	(0.94, 4.79)	8	1.38	(0.60, 2.72)
Lung	[C33-C34]	45	0.92	(0.67, 1.24)	6	1.36	(0.50, 2.95)	15	0.95	(0.53, 1.57)	24	0.84	(0.54, 1.25)
Melanoma	[C43]	11	0.70	(0.35, 1.25)	0	0.00	(0.00, 2.48)	6	1.14	(0.42, 2.48)	5	0.56	(0.18, 1.30)
Prostate	[C61]	83	0.85	(0.68, 1.05)	12	1.35	(0.70, 2.35)	20	0.64	(0.39, 0.98)	51	0.89	(0.66, 1.17)
Bladder	[C67]	16	1.10	(0.63, 1.79)	1	0.79	(0.02, 4.38)	6	1.30	(0.48, 2.82)	9	1.05	(0.48, 1.98)
Kidney	[C64-C65]	19	1.65	(0.99, 2.57)	5	4.64	(1.51, 10.83)	4	1.05	(0.29, 2.70)	10	1.50	(0.72, 2.76)
Brain	[C47; C70-C72]	3	0.73	(0.15, 2.13)	1	2.54	(0.06, 14.17)	0	0.00	(0.00, 2.68)	2	0.86	(0.10, 3.09)
Thyroid	[C73]	1	0.30	(0.01, 1.67)	0	0.00	(0.00, 11.05)	0	0.00	(0.00, 3.21)	1	0.54	(0.01, 3.00)
Haem&Lymph^1)^	[C81-C85; C88; C90-C96; D45-D47]	21	0.78	(0.48, 1.18)	6	2.47	(0.91, 5.37)	6	0.69	(0.25, 1.50)	9	0.56	(0.26, 1.07)
All except NMSC	[C00-C97; D45-D47; exc. C44]	291	0.93	(0.82, 1.04)	41	1.37	(0.98, 1.86)	90	0.86	(0.69, 1.06)	160	0.89	(0.76, 1.04)

^1)^Neoplasms in the haematologic/lymphatic system.

For all cancer sites combined, SIR was estimated at 1.05 (95% CI 0.92, 1.20) for females and at 0.93 (95% CI 0.82, 1.04) for males. However, the risk for all cancer sites combined is prone to a different site mix for females than for males (sex specific entities, different frequencies of some sites like lung or head&neck between females and males) and therefore must be interpreted with caution.

**Figure 1:**
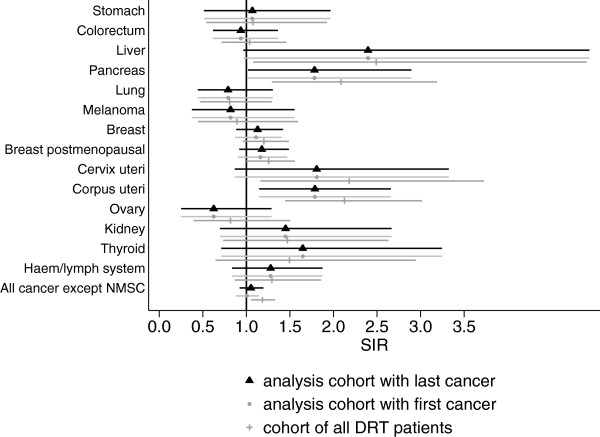
**Sensitivity analysis for female patients, age-adjusted SIR and 95% confidence interval for analysis cohort, analysis cohort with first cancer case and cohort of all patients registered in the DRT (also without date of diabetes diagnosis).**

**Figure 2:**
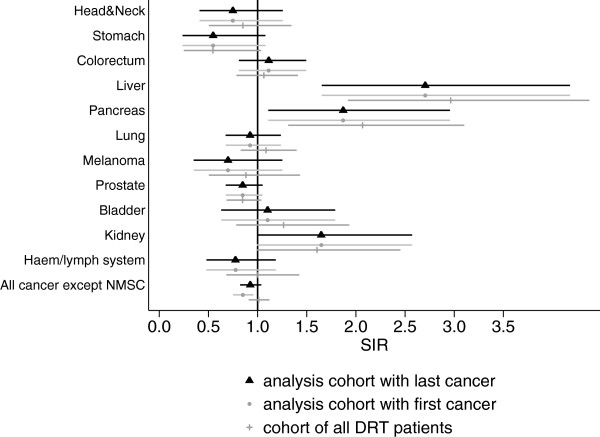
**Sensitivity analysis for male patients, age-adjusted SIR and 95% confidence interval for analysis cohort, analysis cohort with first cancer case and cohort of all patients registered in the DRT (also without date of diabetes diagnosis).**

The results of the sensitivity analysis are shown in Figures 1 and 2. For all sites with statistically significantly elevated SIR the risk was of comparable size for all three scenarios analyzed. However, there was a tendency towards higher SIR estimates in the cohort of all DRT patients, with and without registered date of diabetes diagnosis.

## Discussion

This study combined the data of two Tyrolean registries, the Diabetes Registry and the Cancer Registry, and showed increased risks for liver and pancreatic cancer in both male and female T2DM patients. In addition, the cervix and corpus uteri cancer risk was elevated in females and the kidney cancer risk in males. When analyses by time since T2DM diagnosis were performed the most increased risk for liver cancer was observed after five years in females and after two years in males whereas the increased pancreatic cancer risk peaked in the first year after diagnosis. A similar pattern was detected for cervix and corpus uteri cancer in females and kidney cancer in males.

In general, our results are in agreement with those of epidemiological studies published in the literature. For pancreatic cancer Huxley et al. [2] found a risk estimate of 1.82 (95% CI 1.71, 1.94) in a meta-analysis, and Ben et al. [3] found a slightly higher risk estimate of 2; both estimates fit quite well with our results of 1.78 and 1.87 for women and men, respectively. Ben et al. also reported that the risk for pancreatic cancer diagnosis peaks shortly after diagnosis of diabetes [3]. In our study, cancer diagnosis was highest in the first year after entry in the DRT but risk was increased also after a longer observation period. A meta-analysis performed by El-Serag et al. [24] found a risk estimate of 2.50 (95% CI 1.93, 3.24) for liver cancer in patients with type 2 diabetes, and some years later Wang et al. [2] demonstrated in a meta-analysis a summary relative risk of 2.01 (95% CI 1.61, 2.51). These results are in line with the risk observed in our study, namely 2.40 for women and 2.71 for men. Our results are also in line with further observations in the Wang et al. [2] paper namely that the risk was increased in both females and males and that the risk was stronger in studies with a follow-up period longer than six years. In addition, Wang et al. also showed that increased liver cancer risk for diabetes patients. is independent of alcohol consumption and hepatitis viral infection.

For endometrial cancer Friberg et al. [4] reported a risk estimate of 2.10 (95% CI 1.93, 3.24) as compared to a risk estimate of 1.79 in our study. Also, diagnosis of endometrial cancer in our study was highest within a short period following diagnosis of T2DM and also elevated in the period beyond five years after diabetes diagnosis. Our study also observed an increased risk for diagnosis of cervical cancer, which to our knowledge has been shown only for patients with the diagnosis of type 1 diabetes mellitus [25].

For bladder cancer we found a risk estimate of 0.89 for female and 1.10 for male patients with diabetes mellitus; the latter is in agreement with the results of a previously published meta-analysis showing a risk estimate of 1.24 [8]. With respect to bladder cancer the small number of cases of this cancer entity has to be considered, which results from very strict diagnostic definitions. Additionally, several authors suggest that bladder cancer data can hardly be compared internationally due to the lack of uniform definitions [26].

Our analysis did not show an increased risk for colorectal carcinoma diagnosis in patients with T2DM. We demonstrated risk estimates of 0.94 and 1.11 for females and males, respectively, as compared to a risk estimate of 1.40 shown in a meta-analysis by Larsson [27]. Similar results were published by Jiang et al. [6], Yuhara [28] and Flood [29]. We also found no increased risk for gastric cancer (risk estimates of 1.07 for female and 0.55 for male patients with T2DM), which is consistent with the ongoing discussion about an increased risk for gastric cancer in Western populations [11, 13, 18]. In addition, stomach cancer has decreased markedly in our region and numbers are small; for Tyrol we previously observed an age-standardized incidence risk of 5 and 11 per 100 000 females and males, respectively [22].

Our risk estimate for prostate cancer (0.85, 95% CI 0.68, 1.05) failed to reach statistical significance. However, our SIRs are in the range of those in the meta-analysis conducted by Kasper et al. [10] showing a risk estimate of 0.84.

In summary, our study findings agree in general with those of published studies conducted in hospitalized patients [30].

Causality of the association between diabetes mellitus and cancer is far from being the aim of our study. Some of the most discussed pathways between diabetes mellitus and cancer are common risk factors, for example for endometrial cancer (obesity), pancreatic cancer (smoking, obesity) and breast cancer (dysregulation of female endogenous sex hormones [31]).

Because our evaluation is based on the combined analysis of two registries, no detailed clinical data were available for the patients. The diabetes patients in our study were all treated in hospitals and therefore we hypothesized that our patients are prone to a selection towards more severe course of diabetes. DRT entry was based on data obtained in an out-patient department setting and thus with comprehensive diagnostic tools; results derived from patients treated in private practice might be different.

For most cancer sites with increased cancer risk in T2DM patients, risk was increased mainly in the first year after diabetes diagnosis. For this observation made in many studies on the association between diabetes and cancer two main causes are under discussion, namely reverse causality (this means that in fact cancer causes T2DM and not that T2DM causes cancer, which was the association we intended to investigate) and ascertainment bias (because the diabetes diagnosis is likely to initiate a chain of diagnostic interventions that could result in a cancer diagnosis occurring around the time of diabetes diagnosis, which cancer diagnosis would not have been made at that time without diabetes diagnosis). In general, all studies on diabetes and cancer underlie an inherent ascertainment bias described above. This ascertainment bias was studied in detail, for example by Johnson et al. [32].

Reverse causality was discussed to play a role in the association between diabetes and cancer. For pancreatic cancer reverse causality could be the case, a theory that is supported by the increased risk observed shortly after diagnosis of diabetes. While it is noteworthy that we were able to present data on SIR by time since diabetes diagnosis, our numbers are small and therefore prone to random variation. Risk for pancreatic cancer was higher in the first year after diabetes diagnosis in our study too, but a tendency towards increased risk was noticed also after the immediate period following diabetes diagnosis. Reverse causality seems less likely for liver cancer, because peak values for diagnosis years were observed after T2DM diagnosis. In our study we observed elevated SIR estimates during the whole follow-up period analyzed. We also observed elevated SIR estimates after the first year following diabetes diagnosis for kidney cancer (reaching borderline statistical significance for men). Larsson et al. [7] showed in a meta-analysis of cohort studies a tendency of stronger association in women than in men which is not in line with our observation. In addition, it is unclear whether the observed association between diabetes and kidney cancer risk is due to detection bias.

In addition to reverse causality and ascertainment bias, both disorders can be viewed as chronological diseases with long latency periods and diagnosis at some arbitrary time point depending on clinical symptoms. Johnson pointed out that the clinical conditions leading to diagnosis of diabetes and/or cancer can be overlapping [18].

In summary, the association between T2DM and certain types of cancer seems to be very complex. Differences and limitations in the applied methods make it difficult to compare study results [16, 18, 31]. This is also true for the ongoing discussion about a possible influence of glucose-lowering medication on cancer risk.

In order to investigate the possible influence of the study design on the results of our evaluation, we also performed a sensitivity analysis comparing our results with 1) chronological first cancer (instead of chronological last cancer) and 2) a cohort of all cases collected in the DRT, with and without registered date of T2DM diagnosis. While these analyses reveal no significant differences from the overall study results, there is a tendency towards more elevated SIR estimates in the group of all DRT patients.

Our study has strengths and limitations. The latter are mainly the lack of detailed clinical data, especially data on comorbidities. In addition, it is likely that we underestimated the effect because the standard for computation of SIR was given by the whole population, which includes also T2DM patients at an estimated prevalence of at least 6%. The most severe limitation is that, like all observational studies, also our study is prone to all potential biases inherent in observational studies, lack of confounding factors as mentioned above and also the potential interactions of diabetes and cancer, for example problems involving reverse causality, common risk factors, competing risks, ascertainment bias etc. For a more detailed list see [18]. The distinct strengths of this study are the evaluation based on data from two well established registries operating in the Austrian state of Tyrol: cancer data were collected prospectively and the high quality of the Cancer Registry of Tyrol has been demonstrated [19]. For all diabetes mellitus patients diagnosis was based on clinical data, and all diabetes mellitus patients diagnosed since 2005 were prospectively registered. The observational study was based on well-defined design principles that were set a priori.

## Conclusion

In conclusion, the main finding of this study is that the risk for certain types of cancer is increased in patients with T2DM in this Central European region. Nevertheless, it is important to interpret the results with great caution due to inherent methodological problems. Optimized care programs for patients with T2DM should be integrated in the recommended procedures for cancer screening [16, 33].

## Ethical aspects

The study was approved by the Ethics Committee of Innsbruck Medical University.

## Abbreviations

CI: Confidence interval
CRT: Cancer Registry Tyrol
DRT: Diabetes Registry Tyrol
SIR: Standardized incidence ratio
T2DM: Type 2 diabetes mellitus.
